# What is health? A deconstruction of the World Health Organization 1948 definition

**DOI:** 10.1177/13634593251393743

**Published:** 2025-11-27

**Authors:** Emmie Wahlström, Jonas Stier, Benti Geleta Buli

**Affiliations:** 1 Mälardalen University, Västerås, Sweden

**Keywords:** deconstruction, definition, health, WHO

## Abstract

For 75 years, the WHO health definition has been the most influential and widely accepted definition of health globally, having a profound impact on policy making, professional practice, and people’s overall understanding of health. Although there are alternative notions and definitions of health, it is argued that this definition has enjoyed an almost hegemonic position in the health discourse. Still, it has rarely been systematically deconstructed. This text aims to deconstruct the WHO 1948 definition of health in its entirety as well as its parts. This is done to highlight the epistemological variations and potential contradictions embedded within, and the possible consequences of the definition. The findings indicate a multitude of occasionally ambiguous or conflicting readings depending on how the reader and contemporary ideas conceptualize words and phrasing in the definition. For example, interpreting health as binary can be challenged by ideas of the present as constantly changing, suggesting that health in the definition is conceptualized along a continuum. Such alternative – and sometimes contradictory readings – call for clarifications on how the definition is used and interpreted in different contexts. Still, its ambiguity might have helped it remain relevant and applicable across different societies and time periods.

## Introduction

Formulated in 1948, the World Health Organization’s (WHO) definition of health states that *“Health is a state of complete physical, mental and social well-being and not merely the absence of disease or infirmity.”* Although there are alternative notions and definitions of health, for 75 years, the WHO’s definition has occupied an almost hegemonic position in the health discourse, having a profound impact on policy making, professional practice, research, and people’s understanding of health. Yet, although sometimes criticized (Huber et al., 2011; Leonardi, 2018; Nobile, 2014), the WHO definition has rarely been systematically deconstructed. This paper contributes by providing such a deconstruction, aiming to explore perspectives and meanings embedded within the definition. This is not done to arrive at an all-compassing, exhaustive health definition, but to highlight epistemological variations and potential contradictions embedded within the definition and the possible consequences of these. In doing so, this paper offers a significant contribution to the understanding of the epistemological underpinnings underlying the definition.

### Defining health and the WHO definition

Through the developments in health and medicine over time, the definitions of health have changed. Hippocrates defined health in terms of the relationship between the environment, especially cleanliness, and the occurrence of disease (Yapijakis, 2009), whereas Galen, a second-century AD physician, stressed a holistic approach, considering a patient as “a whole,” including mental and emotional states (Flaskerud, 2012). The discovery of microorganisms in the causation of infectious diseases (Gest, 2004) and the proliferation of experimental science following the discovery of genes (De Castro, 2016; Olby, 1979) also impacted evolving definitions of health. It was with this backdrop within the field of health sciences that the WHO definition of health came to exist (World Health Organization (WHO), 1948).

Adopted in 1948, the WHO constitution laid the foundation for a new definition of health. Rather than equating health solely with the absence of disease or infirmity, a comprehensive and positive perspective was emphasized (Larsen, 2022). With a vision that the enjoyment of health should be a fundamental human right transcending all differences (WHO, 1948), health was redefined to be a holistic state encompassing physical, mental, and social well-being. This redefinition was established as a contrast to how health was defined by the prevailing traditional paradigm of medicine, and was a product of a strong political and scholarly ambition to change current health understandings and health policies (Larsen, 2022).

In addition, in the post-World War II era, establishing an internationally accepted policy document became a central focus (Huber et al., 2011; Leonardi, 2018). This meant outlining basic principles to ensure the happiness, harmonious relations, and security of all peoples (WHO, 1948). It was against this backdrop that the WHO’s health definition came into existence (WHO, 1948). Since 1948, this definition has been used globally and has been cited and praised for its comprehensiveness (Schramme, 2023). At the same time, there has been criticism and frequent calls for its reformulation (Huber et al., 2011; Larsen, 2022; Leonardi, 2018).

### Aim

Given both its profound impact on policy making, professional practice, and research, as well as criticism raised over the years, the WHO 1948 definition of health continues to be used. To understand the reason for this, this paper aims to critically analyze it using a deconstructive approach. This is done by deconstructing the definition in its entirety, examining its constituent parts, and the epistemological variations and contradictions embedded within the wording. By doing so, the ontological and epistemological underpinnings of the definition are brought to light to critically discuss the definition’s content and increase understanding of why the definition continues to be used. Finally, this text is a contribution to the overall, ongoing discussion on how health should be understood in policy making, approached in professional practice, and conceptualized in research.

## Deconstruction as an analytical approach

The epistemological point of departure of deconstruction is to question established notions and facts, the taken-for-granted, to explore what is going on within them while simultaneously reflecting on the process of constructing these explorations (Derrida, 2021). Deconstruction is made up by “*not the mixture but the tension between memory, fidelity, the preservation of something that has been given to us, and, at the same time, heterogeneity, something absolutely new, and a break*” (Derrida, 2021: 6). Deconstruction entails an aspiration to think and rethink, to question and then reframe questions to the constructed answers, to engage with the impossible.

In deconstruction, the concept of time is central, and the focus is on the present as a point of departure. The present needs to be understood with regard to a history of what has shaped the present, while considering that history is formed and reshaped in and by the present (Derrida, 1982). Ideas concerning the present thus introduce the volatility of the present as an always-shifting point in time. The present’s volatility means that the value or definition of something is always “to come” or shifting depending on the time, place, context, and thinker (Derrida, 2021). Similarly, this volatility entails a constant (re)production of words and meanings in the interactions between a text and a reader, allowing for variations in interpretations dependent on person, space, time, etc. Thereby, texts, words, sentences, and arguments are filled with inconsistencies, double-meanings, unspoken intentions, etc., that shape how they may be constructed depending on who is interpreting what is said or written, in which context and time, that is, in what “present” moment.

The focus for deconstruction is the epistemological construction of an “inside” and an “outside” of what is read (Derrida, 1982). The inside is constructed against a backdrop of an outside, defining the inside of *what* is read or centered *in* the reading. By reading, thinking about, and questioning what is centered, the reflection of what is not centered, that is, the outside or opposite, becomes part of what is constructed as the inside. The outside provides presuppositions or taken-for-granted assumptions about the inside. In addition, by their formulation and order, wordings describing something, illustrate a content or an inside as well as an outside. Thus, deconstruction debunks what is centered, its position, the boundaries (separating the inside from the outside), and the space between the inside and outside. For these reasons, the following deconstruction serves as a way of exploring the epistemological relationship between the inside and outside of the WHO definition. By doing so, words used are brought forward, along with the myriad of intended meanings embedded in their usage and combinations.

## Methodology

This study applies a deconstructive approach to the analysis of the WHO definition of health from 1948, which states that “*Health is a state of complete physical, mental and social well-being and not merely the absence of disease or infirmity*” (WHO, 2020). In the analysis, the WHO definition has been divided into sections. Each section has been approached as a whole and worked through word by word to illustrate the multitude of interpretations embedded in the definition and what is left outside of the definition.

The analysis began with BG and EW brainstorming possible interpretations and readings of the sections, one at a time. Based on the brainstorming notes, BG and EW individually analyzed one section at a time. Each section was read through, and alternative readings were explored by relating them to the definition in its entirety, scientific literature, and dictionaries defining the specific words and wording. During this process, BG and EW continuously discussed readings and interpretations. In this phase of the analysis, JS also participated as a critical discussion partner, questioning, and challenging the preliminary interpretations and readings. Based on these discussions, the preliminary descriptions of interpretations and readings were developed into the results by BG, EW, and JS.

When using deconstruction, the authors’ preconceived ideas and notions need to be accounted for. Our 2024 reading of the 1948 WHO definition took place at a different time and place than the one in which the definition was formulated. The analysis was conducted by three researchers within public health science, social work, and sociology. BG is trained and educated in public health sciences in Ethiopia and South Africa, and has practiced public health work in several African countries. EW is educated in public health sciences in Sweden and works within the area of social work. JS is a sociologist and a professor of social work in Sweden and has extensive knowledge within the fields of sociology and intercultural studies. None of us are native English language speakers.

The WHO definition serves as the first principle in the constitution and is followed by eight additional principles that are “*basic to the happiness, harmonious relations and security of all people.*”. Given that no theoretical underpinnings of the definition are presented together with the definition in the WHO constitution, the words, wordings, and structure of the definition have guided the analysis. Yet, the analysis is also shaped by our experiences, knowledge, and biases, and additional readings that are not presented in this analysis might be available. Still, the diverse backgrounds of the research team have contributed to expanding the analysis. Finally, we acknowledge the efforts of the WHO health definition committee to include balanced clinical and non-clinical aspects of health, and we recognize that we are deconstructing a definition adopted over 75 years ago, using today’s perspectives. The passage of time brings historical changes in all aspects of life, and thus, our understanding of health evolves continuously.

## Results

The presentation of the results will follow the structure of the definition and the approach used in the analysis. The definition, “*Health is a state of complete physical, mental and social well-being and not merely the absence of disease or infirmity*” (WHO, 2020), is divided into five sections starting with “*Health is a state. . ..*”

### Health is a state. . .

In the constitution, the definition starts by centering on health. Health is used as an overarching concept and not linked to any particular being (i.e. humans in general, a particular individual, or animals). Yet in the subsequent text, it is posited that health is located within an individual: “*. . . health is one of the fundamental rights of every human being. . .*” (WHO, 2020: 6). Locating health within *every* human being constructs boundaries for whose health the definition relates to and conceptualizes health as a human-centric concept rather than one shared by all living beings.

Following the word “health” in the definition is the word “state,” as in “health is a state. . ..” The inclusion of “state” has been argued to be problematic (Bickenbach, 2015; Leonardi, 2018; McCartney et al., 2019; Schramme, 2023), primarily due to how the word “state” conceptualizes health as a binary or static phenomenon (health vs no health) (Huber et al., 2011; Leonardi, 2018; Oleribe et al., 2018). Such a reading, where health is binary and static, is common, yet state may also be read as a shifting point on a scale. Such an alternative reading may depart from Derrida’s descriptions of the present (Derrida, 1982), where the now or present is constantly shifting. Given this perspective on the present, “a state” changes with every new now; it is always in a condition of becoming. By understanding “a state” as only located in the present, fluctuation of the “state” becomes possible as the present is constantly changing. Schramme (2023) similarly argues that the definition was formulated to allow for fluctuation of health, given the disciplinary backgrounds of the authors of the declaration in public health. Yet, prior criticism of the definition tend to position the binary reading of state as the “norm,” thereby constructing readings suggesting a more fluid or fluctuating interpretation as a constitutive outside.

Another alternative reading of state, which is also used as an argument in criticism of the WHO definition (Huber et al., 2011; Leonardi, 2018), departs from the word being read as a social position, as in either an elaborate or luxurious style of living or formal dignity (Merriam-Webster, n.d-g). Departing from such a reading, Huber et al. (2011) and Leonardi (2018) both argue that the use of state implies that health is something only achievable for a few (the fortunate and affluent), if any, of the global population of human beings. This criticism also builds upon understanding state as a condition of “abnormal tension” (Merriam-Webster, n.d-g), not something experienced in everyday life. This reading relates the understanding of state as both binary and static, as well as fluctuating, while implicitly constructing health both as the endpoint of a scale and as an ideal to strive for.

A third alternative reading may illustrate state as a condition of being (Merriam-Webster, n.d-g), or more precisely, a condition of mind or temperament or a condition or stage in the physical being of something. This reading thus focuses on a condition of a body or an entity. Departing from this reading, the definition would suggest that health is a condition of mind and temperament (psychological aspects) and or of the physical being (physical aspects). This suggests that both psychological and physical aspects would be central when determining the body’s condition, while simultaneously presenting a division in what should be regarded when health is determined. Both the focus when determining the condition and the division of body and mind in this reading suggest that this third alternative reading might underline an argumentation of the definition being shaped by a (Western) medical paradigm. Although previous criticism of the definition has rarely focused on the taken-for-granted nature of the medical paradigm’s construction of health embedded in this third alternative reading, there has been related criticism. This criticism has mainly focused on claims of society’s medicalization (Huber et al., 2011; Leonardi, 2018), where arguments concern how the inclusion of multiple health dimensions in the definition would suggest that any “problem” linked to poor health is assumed to have medical roots and medical solutions. Such arguments also include that the definition can elevate the medicalization of social or economic causes of poor health (e.g. social injustice or poverty) (Bickenbach, 2015) and thereby redefine the boundaries of health and disease (Huber et al., 2011). Still, this reaffirms the taken-for-granted nature of the (Western) medical paradigm’s division of mind and body and positions other holistic perspectives on health outside of the definition (McKee, 1988; Seidlein and Salloch, 2019).

A fourth reading of state becomes available when reading the phrasing in which state is embedded, that is, “*Health is a state of*.. . .” The phrase presents health as a state of something, in this case, (1) well-being; and (2) absence of disease or infirmity, implying that state and result are synonymous. Understanding health as a result of several factors is supported by the third reading, suggesting that state is a condition of psychological or physical factors and departing from a medical paradigm. Still, this fourth alternative reading also broadens the idea of this state’s constituent factors. In the subsequent text in the constitution (WHO, 2020: 1), multiple factors are described to impact health. Health is described as dependent upon the collaboration of individuals and states, a result of peoples’ medical, psychological and related knowledge, of informed opinion, active cooperation, and of the work of governments. Yet, the subsequent text also introduces additional descriptions of what the state is. These describe health as “*fundamental to the attainment of peace*” and “*basic to the happiness, harmonious relations and security of all peoples*” (WHO, 2020: 1), suggesting that the state (i.e. health) is not only a result but an essential part of a process to achieve something else. Thus, reading the WHO definition as part of the WHO constitution suggests that a state is a dual position – both a result and part of a process. Subsequent documents by WHO support such a reading of state. For example, the health definition of 1986 describes the state (i.e. health) as “a resource for everyday life, not the objective of living” (WHO, 1986: 5). By doing so, the state is described as an essential part of a process to achieve something else. However, the WHO definition of 1986 also affirms that the state is a result by describing that it is dependent on an individual’s or group’s ability to identify and realize “aspirations, satisfy needs and change or cope with the environment” (WHO, 1986: 5).

Given the four alternative readings of state, which all have been applied to varying degrees by others, it may be suggested that the definition lacks clarity in how “state” is expected to be understood. Still, in the documents by WHO, the state is presented as a result of various aspects, and a result that is an essential part of a process to achieve something else. It may be suggested that readings departing from a (Western) medical paradigm are common, thus positioning these as the inside of the definition.

### . . . of complete . . .

The definition continues by introducing the word “complete,” stating that “*health is a state of complete. . .*.” The inclusion of “complete” is one of the most commonly criticized elements of the definition (Huber et al., 2011; Leonardi, 2018; Oleribe et al., 2018), Yet, the importance of “complete” may be questioned as the inclusion has been claimed to be merely an editorial decision (Schramme, 2023). Most of this criticism departs from reading “complete” as “total or absolute,” “brought to an end” or “highly proficient” (Merriam-Webster, n.d-c), which provides grounds for criticism in that the definition disregards the life course variations of a person’s health and overemphasize a state of absence of disease or infirmity and complete well-being (Huber et al., 2011; Leonardi, 2018; Oleribe et al., 2018). Similarly, arguments are made that “complete” depicts health as an unachievable goal, rendering most people unhealthy for most of their lives and excluding the growing number of people suffering from chronic diseases and disabilities from ever attaining health (Huber et al., 2011). In addition, “complete” is argued to disregard the fact that the absence or loss of well-being might not be a sign of poor health but an effect of adequate and realistic reactions to external conditions or unexpected events.

Still, Schramme (2023) presents an alternative reading of “complete” based on defining it as “having all the necessary parts, elements or steps” (Merriam-Webster, n.d-c). Schramme (2023) argues that including “complete” in the definition was not intended to be understood as striving for perfection in a utopian sense but was a way to summarize various dimensions of well-being (physical, mental and social) as a foundation of health. Given that the WHO definition states that “*Health is a state of complete physical, mental and social well-being. . .*” a reading based on understanding “complete” as having all the necessary parts implies that the three dimensions of well-being constitute the necessary parts that construct the state (i.e. health). Yet, this alternative reading of “complete” has been seen in criticism arguing that the breadth and inclusion of multiple well-being dimensions make the definition too complex (Leonardi, 2018). The criticism targets what constitutes a necessary part by questioning the ambition of including medical, environmental and existential aspects, problems pertaining to living conditions, as well as moral and political perspectives on conditions vital for people’s well-being. Similarly, the focus on necessary parts also questions how the last section of the definition – the *absence of disease or infirmity* – might be understood. Based on this alternative reading, the inclusion of “complete” makes a distinction between the necessary parts and other parts of the state. Such a reading relates to intentions communicated during the work leading up to the definition where “*complete physical, mental and social well-being*” was first emphasized in order to shift focus from the prevailing medically influenced understanding of health at the time (Larsen, 2022) represented in “*not merely the absence of disease or infirmity*.”

### . . . physical, mental and social well-being . . .

The following part of the definition introduces the concept of well-being by relating it to health: “*Health is a state of complete physical, mental and social well-being. . ..”* The phrasing suggests that well-being is what constitutes health, yet later definitions of well-being by the WHO challenge such a reading. WHO (2021) defines well-being as “*a positive state experienced by individuals and societies. Similar to health, it is a resource for daily life* . . .,” thus suggesting that well-being and health are two separate concepts, and are not necessarily interlinked. Departing from a reading where well-being constitutes health is also challenged by lexical definitions of well-being that use health as a concept describing well-being: “*the state of being happy, healthy, or prosperous*” (Merriam-Webster, n.d-h). The relationship between health and well-being thus becomes more complex when reading how well-being is defined. Given this complexity, possible readings of the definition include – health is a state of physical, mental and social positive states experienced by individuals and societies (departing from WHO, 2021) and – health is a state of physical, mental and social health, happiness and prosperity (departing from lexical definitions). These readings highlight challenges because the constituting parts (i.e. physical, mental, and social dimensions of well-being – also known as health) and what is defined (i.e. health – also known as well-being) become hard to differentiate. Still, these challenges are not often voiced in criticism of the definition.

Instead, the criticism has focused on the interconnectedness of the three dimensions of well-being and how it resonates with “complete,” as a change in one or more of these dimensions can affect the status of the other dimensions (Leonardi, 2018). Similarly, the definition has received criticism for its epistemological imprecision regarding its components, and measurement and assessment challenges (Huber et al., 2011; Oleribe et al., 2018). These criticisms relate to the variation in possible readings of how the interrelationships can be understood in the definition. One reading of the interrelationships may depart from understanding the use of the conjunction “and” in *physical, mental and social well-being* as a connection of items of the same class or type (Merriam-Webster, n.d-b). This reading would thus suggest that the three dimensions are equally important and integral to achieving a state of complete well-being. The equal importance could be read as derivable from the sum of the individual dimensions of well-being (referring to the adaptive approach by Abrams et al., 2020). However, this implies that all dimensions need to be at a maximum to achieve health, which (as previously discussed) might not be practically applicable to an individual’s health. A reading departing from an intersectional approach (Abrams et al., 2020) would suggest that the interaction between components goes beyond the additive approach and may account for occasions when, for example, positive social relationships bolster mental well-being without resulting in physical well-being. Yet, neither of these approaches addresses completely how a reading of the definition could relate to issues of objective and subjective measures and possible differences between the two.

Still, the interconnectedness could also be understood based on an assumption that the order in which the dimensions are presented shows their importance, given that they are not presented alphabetically. The order in the definition implies that the physical dimension is the most important and the social dimension the least important. Such an assumption would also imply that a weighted composite approach could be applied to account for the significance of each dimension’s contribution to health (Baldwin, 2015). This alternative reading is consistent with arguments by Stoewen (2017) and reflects the emphasis on granting primacy to physical well-being that prevailed at the time. Still, it should be noted that while the three well-being dimensions are widely acknowledged, regardless of their order, their inclusion constitutes the inside of the definition. Considered separately they could be read as three different dimensions aiming to complete a whole. The inclusion of these three dimensions also renders questions about what is left outside of the definition. The spiritual dimension is for example also regarded as important for health and well-being, (Dhar et al., 2013; Larson, 1996; Tabei et al., 2016) but maybe not important enough to be included in the definition. This suggests that the WHO definition’s centering on the three included dimensions of well-being has become hegemonic (Holst, 2020), where other health aspects are viewed as less important compared to the ones already included in the definition.

### . . . and not merely . . .

The definition’s phrase “*and not merely*” bridges two of its main components: “*a complete state of physical, mental, and social well-being*” and “*absence of disease or infirmity*.” One reading of this phrase suggests that both components hold equal importance and truth, given that “not merely” can denote two equally true conditions (Merriam-Webster, n.d-f). Following this assertion, “a complete state of physical, mental, and social well-being” and “absence of disease or infirmity” both share equal value for achieving health. Yet, acknowledging that these are two equally true conditions represent a contrast between the absence of disease or infirmity and the presence of well-being. The contrast can be read as a binary, complicating measurability and practical application (Huber et al., 2011; Oleribe et al., 2018), as well as pursuing a need to embrace a continuum of health states (Krahn et al., 2021; Sartorius, 2006). An alternative reading could propose that “not merely” is used before the less important of two contrasting truths to emphasize the more important one (Collins Dictionary, n.d.). That said, the statement preceding “and not merely” – that is, “*a complete state of physical, mental, and social well-being*,” is considered more important than “*absence of disease or infirmity*,” which follows the phrase. Such a reading suggests that the former is more significant, an argument which is in line with the importance of including well-being in the definition to position the definition in relation to other declarations (Larsen, 2022). Placing the well-being part first could thus be read as accentuating this new health dimension, contrasting it with the notion that the definition medicalizes problems (Bickenbach, 2015), yet it is also argued that the inclusion of well-being was mainly political and was less important for defining health (Larsen, 2022). Still, a third reading of the phrase “and not merely” may not place the same emphasis on order but rather suggests a holistic approach to health where health, as complete well-being, is more expansive than the objectively measured disease or infirmity (Liamputtong et al., 2012).

### . . . the absence of disease or infirmity

The definition’s last section completes the sentence by linking the state of well-being to “*the absence of disease or infirmity*”. By its focus on absence, disease and infirmity, one reading might suggest that this section reflects a biomedical paradigm of objectivity, measurement, and diagnosis. The biomedical underpinning could be detected through the inclusion of “disease,” rather than “sickness” or “illness,” other concepts denoting poor health. Disease relates to objectively measured or diagnosed dysfunctions or deviations of the body or mind (Boyd, 2000). By contrast, sickness can be explained as a social role, status, or position interlinking with social categorizations and representations of what is expected of “the sick,” whereas illness refers to feelings of being unwell or unhealthy. The inclusion of “disease” over illness or sickness could thus be read as a focus on the absence of objectively detected dysfunctions or deviations, highlighting both a focus on detection by an external party and deviations from an assumed normality. It may also imply that only people whose health statuses have been diagnosed or assessed by a health professional can be declared or defined as having an absence of disease or infirmity (i.e. being healthy). Consequently, conditions impacting the health or feelings, not yet diagnosed by a health professional as unwell, are not seen as valid when determining that person’s health. In addition, “absence” could also be read as underlining the complete lack of disease or infirmity, further implying a binary interpretation of health. This reading of “absence” reaffirms the biomedical perspective on health that is given in the inclusion of disease in the definition, and contributes to constituting the biomedical paradigm as the inside shaping this latter part of the definition.

The last part of this section, “. . . or infirmity,” could also be read as relating to objectivity and normality as infirmity relates to weakness explained as *the quality or state of being infirm* (Merriam-Webster, n.d-e), that is, *“1) of poor or deteriorated vitality, especially feeble from age, 2) weak of mind, will or character: irresolute, vacillating, or 3) not solid or stable: insecure*” (Merriam-Webster, n.d-d). In such a reading, the weakness is implicitly related to a normality against which the poorness of vitality, weakness and lack of stability are measured. Weakness, on the one hand, is related to mental (i.e. mind and will) or character-based (*“a personal failing: foible*” (Merriam-Webster, n.d-e) flaws or shortcomings (psychological aspects). On the other hand, weakness is related to a lack of strength (*the condition of being feeble: frailty* (Merriam-Webster, n.d-e) and vigor (vitality, solid and stable) (physiological aspects). This presents a reading where the last section “*the absence of disease or infirmity*” could be read as – the absence of disease or weakness in a psychological and or physiological sense. This might to some extent resonate with a biomedical paradigm of objectivity and normality, but lacks precision when it comes to measurement and differentiation between what is disease and what is weakness. Yet, infirmity also allows for an alternative reading, where it is understood as weakness due to aging. Such a reading of infirmity would imply that health is dependent on an absence of age-related weaknesses. Given that the aging process leads to “natural” weaknesses of the mind and body, this reading would suggest that health cannot be attained by most people of a higher age (the elderly). Still, this reading reaffirms previous criticism of the definition stating that life course variations are not considered (Huber et al., 2011; Leonardi, 2018; Oleribe et al., 2018), although it departs from another section of the definition.

Returning to the last section as a whole, the phrasing “*. . . the absence of disease or infirmity*,” may, as discussed, be read as the section reaffirming a biomedical perspective on health, yet this reading produces questions in relation to measurement and assessment (Huber et al., 2011; Oleribe et al., 2018). Questions particularly arise in relation to the conjunction “or,” which connects disease and infirmity. This connection implies two possibilities or alternatives for what must be absent (Cambridge University Press, n.d.); either an absence of disease, or an absence of infirmity. A reading departing from this understanding would thus suggest that the absence of only one of the two is sufficient to fulfill this section of the definition. Consequently, someone with an infirmity (i.e. a psychological or physiological weakness), while simultaneously not having a disease would be understood as fulfilling the criterion of absence of disease or infirmity. However, in readings of the definition, this distinction is rarely made, and the “or” is often read as an “and,” suggesting that both disease and infirmity would need to be absent. Such a reading – exchanging the “or” for “and” – have attracted criticism (Bickenbach, 2015; Leonardi, 2018; McCartney et al., 2019; Schramme, 2023). A reading that focuses on the “or,” thus poses new questions as to how the definition should be interpreted in relation to absence of one (e.g. disease) but not the other (e.g. infirmity).

An alternate reading of the last section may depart from understanding absence as a shortage of something expected to be there: “*a state or condition in which something expected, wanted or looked for is not present or does not exist*” (Merriam-Webster, n.d-a). Reading the start of the section would then suggest that the definition implies that disease or infirmity are what is expected, wanted, or sought, that is, the normal state. By suggesting so, the definition departs from a standpoint where people are expected to live for most/all their lives with diseases or infirmity, that is, health is not a baseline in people’s lives; disease or infirmity is. As mentioned, this relates to the criticism from a life course perspective (Huber et al., 2011; Leonardi, 2018; Oleribe et al., 2018), yet it also challenges the criticism that takes for granted that the definition (should) depart from viewing health as a baseline and disease or infirmity as limiting aspects in people’s lives.

Still, the prevailing reading of the last section seems to depart from understanding absence of disease or infirmity as grounded in the biomedical paradigm, where the body and mind are assessed objectively (i.e. by the medical professions), and with normality as the point of reference. This reading constitutes objectivity and normality as the inside of the definition guiding the understanding of what is absent. Consequently, the reading also constitutes a Western medical perspective on health and poor health as the inside of the definition (Seidlein and Salloch, 2019). Left outside the definition are thus the subjectivity in patient experiences of illness when disease is not present, the subjectivity of professionals, and structural or cultural influences in the construction and assessment of diagnoses and medical criteria for diagnosis. Still, the subjective dimension is included in the definition, but only in the part on well-being.

## Concluding remarks

Using deconstruction, this study has endeavored to deconstruct the 1948 WHO health definition, analyzing its components and their interrelationships. This endeavor is important as the definition, since its formulation, has been extensively used and cited all over the world (Holst, 2020), possibly due to the prominence, authority and influence of the WHO. That said, the definition has profoundly shaped research, practice, and policies (Krahn et al., 2021). However, it has also been criticized. While the original definition was constructed in a specific historical and philosophical context, contemporary understandings of health may require more (Leonardi, 2018; Zuckerman et al., 2014). Over the years, the WHO has revisited its health definition, with notable efforts in 1986 and 2001 (McCartney et al., 2019; WHO, 2001), yet no fundamental changes have been made – although any definition needs to adapt to the dynamics of historical and cultural contexts. This adaptability ensures the definition’s relevance and applicability across societies and periods. As the definition has remained unchanged for more than 75 years, there is a dissonance between its idealized notion of health and the contemporary realities of its implementation contexts (Badash et al., 2017).

Similarly, the WHO’s definition of health also suffers from an inherent limitation in being a simplification of reality (Lissack, 2016). Such simplifications, while necessary, also shape reality through their language. As the findings show, the language used in the definition allow for different readings, with different implications for how the words should be understood separately and as a whole. One reading suggests binary views of health, well-being, disease and infirmity. Although the definition is divided into a well-being part and a disease or infirmity part, another reading suggests that the relationship between the constituent words in each part as well as between the parts is not evident. One interpretation suggests that the order of parts and words is irrelevant. Similarly, such an interpretation could also be implied for the components of well-being (physical, mental, and social). However, considering well-being versus the absence of disease or infirmity, the order becomes significant, with the element preceding “and not merely” assuming greater importance.

In summary, while the WHO’s 1948 definition has been highly influential and widely adopted, the multiple readings of it in this deconstruction analysis show a lack of clarity. Although short, the definition exhibits several biases (e.g. the dichotomy of well-being and absence of disease), which might present challenges if it is used as a basis for agreeing on what health should be, how it should change, or how it should be studied. The definition also contains multiple perspectives on health, some of which stress a biomedical perspective, suggesting that health is measurable and objective. Thus, when using the definition, it might need to be accompanied by a clarification about its application and interpretation in different historical, cultural, and social contexts. This underscores the need to be mindful of how definitions impact practice and policy. That said, this paper has made a valuable theoretical – and from a broader professional perspective, professionally relevant – contribution to the understanding of the epistemological underpinnings of the definition.
